# The Role of Nurse Practitioners in Surgical Settings Across the Perioperative Trajectory: A Comparative Study on Patient-Centered Outcomes †

**DOI:** 10.3390/nursrep15080291

**Published:** 2025-08-09

**Authors:** Limor Chen, Ziv Gil, Nasra Idilbi, Dafna Zontag, Efrat Shadmi

**Affiliations:** 1 The Cheryl Spencer Department of Nursing, University of Haifa, Haifa 3103301, Israeleshadmi@univ.haifa.ac.il (E.S.); 2 Rambam Medical Center, Haifa 3109601, Israel; 3 Holy Family Hospital, Nazareth 1641105, Israel; 4 Department of Nursing, Max Stern Yezreel Valley College, Emek Yezreel 1930600, Israel; 5 Galilee Medical Center, Nahariya 2210001, Israel; 6 Azrieli Advanced Nursing Center, University of Haifa, Haifa 3103301, Israel

**Keywords:** surgical unit, nurse practitioner, patient-reported outcome measures, anxiety, pain, perioperative care, elective surgery

## Abstract

Nurse practitioners (NPs) are increasingly integrated into surgical care teams, complementing traditional surgical roles. However, the relationship between their involvement and patient-reported outcome measures (PROMs), such as pain and anxiety, remains understudied. **Purpose**: To examine the types of care from NPs in surgical units during the perioperative period and evaluate their association with length of stay, pain, and anxiety. **Methods**: Our prospective comparative study in two surgical units at a tertiary medical center included 315 patients: 156 received care from NPs, and 159 received usual care. Data were collected at three time points: post-operative day one (T0), during hospitalization (T1), and 14 days post-discharge (T2). Measures included the Brief Pain Inventory, the Hospital Anxiety and Depression Scale, and an intervention checklist completed by the NPs. **Findings**: NPs performed primarily in-hospital interventions including care coordination (40%) and medication management (44%). Patients treated by NPs reported significantly lower in-hospital anxiety compared to usual care (*p* = 0.001). The length of stay and pain levels were not significantly associated with NP care. **Discussion**: NPs in surgical settings provide patient-centered care associated with lower in-hospital anxiety. Further research is recommended to validate these findings in diverse settings.

## 1. Introduction

The aging of the population and increasing complexity of patients’ health conditions are placing significant pressure on healthcare systems, affecting the ability to provide high-quality, efficient care. These demands have sparked the emergence of novel professions that complement traditional healthcare roles [1]. In response to these challenges, healthcare teams have expanded to include professionals with complementary roles, such as nurse practitioners (NPs). NPs provide additional services that supplement physician care [2]. For example, they perform activities such as handling urgent issues, delivering services in person, and examining patients when the primary attending physician is not available to do so. This collaborative approach of NPs complementing physicians’ roles aims to improve quality, efficiency, and continuity of care in surgical settings. For example, a study on the addition of an NP to an inpatient surgical team resulted in overall improvement in the use of resources and reduction in unnecessary emergency department visits [3].

NPs are highly trained professionals who provide advanced nursing care that spans the entire perioperative trajectory. Their role may include assisting in surgery, managing wound care, ordering diagnostic tests and consultations, managing medications, and providing follow-up care to support appropriate recovery [3,4]. In Israel, NPs specializing in surgical care undergo a one-year advanced training program designed for nurses who hold a graduate degree and have significant clinical experience. They independently manage most clinical responsibilities related to surgical care, excluding the performance of actual surgical procedures. Their role focuses primarily on preoperative and postoperative care, including patient assessment, preparation for surgery, follow-up care, and coordination within the surgical team. Since its establishment in 2017, the program has trained five cohorts, resulting in approximately 70 certified surgical NPs to date [5].

International research shows that NPs contribute to the delivery of timely care, addressing the critical need caused by high operating-room volumes and the unavailability of surgeons within inpatient wards. For example, the introduction of a full-time NP to an orthopedic trauma center resulted in improved communication related to discharge planning and to more timely ordering of physical therapy and social services, which otherwise were often delayed until after operating hours [6]. Furthermore, NPs share the workload with other team members and decrease the risk of omissions in care through supervising, reviewing, and disseminating test and procedure results to appropriate personnel, and assuring that discharges are carried out in a timely fashion [7]. A study examining the roles and interprofessional practice of 46 NPs and their team members across nine hospital sites in Ontario, Canada, found that NPs enhance interprofessional collaboration by actively seeking consultation with a diverse range of professionals and by delivering holistic patient care that extends beyond traditional nursing boundaries [8].

Research demonstrates that NPs in surgery settings not only deliver more efficient care but also significantly improve effectiveness by employing a holistic approach and streamlining care processes. A study of implementation of NP roles in a district general hospital in England demonstrated that they are viewed by a range of stakeholders as associated with positive patient experience, outcomes, and safety, even when substituting for a junior doctor [9]. In the emergency department, research has demonstrated that NPs can be as effective as physicians and, in some instances, more efficient, particularly in reducing unnecessary diagnostic services and procedures, and in decreasing the probability of hospital admissions [10]. Additionally, their presence allows resident surgeons to maximize their education by providing them more opportunities to participate in surgical and clinical duties [6,11]. Moreover, the incorporation of NPs in surgical units was found to be associated with better outcomes, including shorter length of stay, lower costs [2], and even lower mortality rates [12,13].

Nonetheless, few studies have examined the contribution of NPs to patient-reported outcome measures (PROMs). This gap is particularly notable given that NPs often spend more time with patients and are perceived as more attentive to their overall well-being, rather than focusing solely on clinical or system-driven objective outcomes [14]. PROMs help identify the challenges faced by patients, inform treatment decisions, and improve adherence [15]. They also offer insight into how healthcare providers influence patients’ recovery experiences, promoting more holistic and person-centered care. Specifically, pain and anxiety are central concerns for surgical patients, influencing both recovery trajectories and overall satisfaction with care. Despite the relevance of these outcomes, evidence on the potential role of NPs in improving pain and anxiety management remains limited [16,17,18].

Our study aims to fill these gaps and to describe the types of care provided by an NP during the perioperative period: at the pre-operative phase, throughout the hospital stay, and in preparation for and immediately after discharge. Additionally, we prospectively evaluated the relationship between NP care, length of stay, and PROMs, including lower levels of pain and anxiety. We hypothesized that there would be differences in reports of levels of pain and anxiety, as well as in length of hospital stay, between patients receiving care from an NP and those treated in similar units without NP presence.

## 2. Materials and Methods

### 2.1. Study Design

We conducted a prospective comparative study in two general surgical units within a tertiary medical center. Due to organizational considerations that preceded the study, one surgical unit (Unit A) employed an NP, while the other (Unit B) did not. Clinical and administrative healthcare data were collected, alongside patient surveys administered in Unit A to those who received NP-led care and compared to patients treated in Unit B without NP involvement. Questionnaires were administered on the day following surgery (baseline) and again 14 days after discharge.

### 2.2. Study Setting and Participants

The study was conducted in two surgical units at a large university-affiliated hospital in Israel between 2022 and 2023. It encompassed patients under the care of an NP in unit A and comparable controls in unit B.

During the data collection period, a trained reviewer screened the daily surgery schedules in both units to identify patients who met the predefined inclusion criteria. Eligible patients were approached on the day after surgery and invited to participate. Recruitment was performed consistently across the study period and across both surgical units.

We included patients with common and specific types of elective surgeries such as hernia repair, bariatric surgery, and gastrointestinal surgeries (cholecystectomy and appendectomy). Participants were eligible if they were hospitalized for elective surgery, were over the age of 18 years, and were cognitively intact. Patients who were terminally ill or unable to speak Hebrew were excluded. The sample size for the prospective arm of the study was calculated using G*Power3.1.9.7, with an alpha level of 0.05 and a statistical power of 0.80. A total sample of 120 to 150 participants across the two surgical units was estimated to be sufficient to detect a mean difference of 1.5 points in HADS anxiety scores between groups, particularly in relation to the quality of the discharge process.

### 2.3. Measures

The Brief Pain Inventory (BPI) [19] was used to assess the severity of pain and its interference with functioning, using the Pain Severity and Pain Interference sub-scales. The BPI was previously translated and used in prior studies with Hebrew speaking populations [20]. In this study, the Cronbach’s alpha coefficients for pain severity in the hospital was 0.84 and after discharge was 0.91, and pain interference in the hospital was 0.77 and after discharge was 0.94. The Hospital Anxiety and Depression Scale (HADS) was used to determine levels of anxiety [21]. For this study, we used HADS Anxiety (HADS-A). Each item is scored on a 4-point scale (0 = not at all; 3 = nearly all the time). The HADS was previously translated and used in studies with Hebrew speaking populations [22,23] The Cronbach’s alpha for HADS-A in hospital and after discharge in this study was 0.93. A checklist was developed to document interventions performed by the NP. This list was produced based on the activities NPs are authorized to perform according to the qualifications of their roles, as determined by the Nursing Division of the Ministry of Health [24]. and through discussion with experienced NPs about interventions performed as part of the role.

The NP reported on all types of activities performed during the care rendered for each patient included in the study, including pre-operative care, post-operative care performed at the unit during the hospital stay, discharge preparedness, and discharge follow-up. Pre-operative care involves coordinating services, such as CT scans, ultrasounds, or biopsies, as well as providing training and guidance to patients and ensuring that all aspects of pre-operative preparation are completed (such as performing diagnostic tests and medication adjustments). Post-operative care encompasses interventions like ordering tests and consultations, managing medications, and conducting daily follow-ups. Discharge-related interventions include discharge counseling for the patient and families, preparing the discharge letter, and performing telephone follow-ups. These interventions span both advanced and traditional nursing interventions. Additionally, data on patient demographic and clinical characteristics and length of stay were collected from the hospital’s electronic database warehouse.

### 2.4. Data Collection

Survey data were collected using Qualtrics, a secure web-based survey platform accessible only to the research team through login credentials. Data were gathered at three distinct time points: at Time 0 (T0), the patient was approached to complete baseline pain and anxiety questionnaires during a face-to-face interview with an external interviewer during hospitalization, the day after surgery. At Time 1 (T1), data was collected from the NP, who completed a checklist of the specific interventions performed for each participant. At Time 2 (T2), questionnaires were administered to patients 14 days post-discharge by telephone interview.

### 2.5. Data Analysis

Statistical analyses were conducted using SPSS version 26. We prospectively examined differences between surgical units A and B (with and without an NP, respectively) for in-hospital and post-discharge pain and anxiety levels. Missing data did not exceed 5% of questionnaire scales. We therefore handled missing data by simple imputation. To examine differences between patients in unit A and unit B, we used independent-sample *t*-tests for continuous variables (e.g., pain scores, anxiety levels, length of stay) and chi-square tests for categorical variables (e.g., sex, surgery type). Multivariable linear regression analyses were performed to test the study hypotheses, controlling for variables which were found to be statistically significantly associated with the study outcomes at the bivariate level, including potential confounders, such as age, sex, and type of surgery.

### 2.6. Ethical Considerations

Ethical approval was obtained from the institutional review boards of both the university (303/21) and the hospital (0050-21-NHR). All participants were informed about the voluntary nature of their participation and their right to withdraw from the study at any time. Data collection was performed after obtaining participants’ written informed consent.

## 3. Results

### 3.1. Demographic Characteristics of Study Participants

We approached 467 patients, of whom 410 (88%) agreed to participate and completed baseline data. Follow-up questionnaires were completed by 315 patients (77%). Patients who completed follow-up data were not different in demographics (sex, age, ethnicity, education) from those who did not complete follow-up. Table 1 presents the baseline characteristics of the overall sample and compares patients who received care from an NP to those in a unit without NP care. Significant differences were observed between the groups in terms of age, sex, education, and type of surgery.

### 3.2. Perioperative Activities Performed by the NP

The activities performed by the NP are described according to the three perioperative categories, relating to pre-operative care, in-hospital care, and post-hospital care (Table 2). The NP was mostly engaged in performing in-hospital care interventions, including coordination of services (n = 127, 40% of patients), consultation orders (n = 82, 26%), and medication management (n = 139, 44%). The second most common category was pre-operative interventions, mostly pre-operative preparations (n = 136, 43%) and coordination of services (n = 109, 34%). Post-hospitalization-related interventions were rarely performed, with the exception of discharge preparations (n = 94, 30% of patients).

### 3.3. Length of Stay and PROMs

Significant differences in outcomes were observed between patients cared for by a NP (Unit A) and those who were not (Unit B) (Table 3). Specifically, patients in Unit A reported lower pain interference after discharge, with a mean score of 0.89 (SD = 1.44) compared to 1.21 (SD = 1.78) in Unit B (*p* = 0.02). Additionally, in-hospital anxiety levels were significantly lower in Unit A patients, averaging 3.52 (SD = 4.37) versus 5.42 (SD = 5.06) in Unit B (*p* < 0.001). Length of hospital stay was also shorter in Unit A by an average of 0.87 days (*p* < 0.001).

The results of the multivariable linear regression analyses (Table 4) show that the difference between the outcomes of patients with and without an NP remained significant only for in-hospital anxiety levels, controlling for all the above cofounders. An additional correlate of less in-hospital anxiety was undergoing hernia surgery (vs. gastrointestinal surgery). Post-hospitalization pain interference was significantly related to older age and having less than 12 years of education (vs. 12 years or more). Longer length of stay was associated with older age.

## 4. Discussion

Our study uniquely examined the relationship between perioperative care provided by an NP and PROMs, including pain and anxiety levels. The results show that employing an NP in a surgical unit is significantly associated with lower in-hospital anxiety compared to care without an NP, while no significant association was found with pain levels. The difference in-hospital anxiety scores between the groups was not only statistically significant but also clinically meaningful, exceeding the minimum clinically important difference (MCID) reported in previous studies among surgical patients [25,26]. While the presence of NPs has been previously associated with lower patient anxiety levels [27], evidence from surgical settings remains limited. For example, a study of vascular surgery patients reported a greater reduction in anxiety levels in those treated by an NP under surgeon supervision compared to surgeon-only care [18].

Our prospective analyses also indicated lower post-discharge pain interference among patients receiving NP care. However, these differences were not statistically significant after adjusting for known risk factors, such as age and type of surgery.

Similarly, a tend toward shorter length of stay with NP care was observed, although it did not reach statistical significance in the multivariable analysis. Nonetheless, accumulating evidence supports the potential for increased efficiency through the employment of NPs in acute care units [11,13]. Similarly to recent findings from other Israeli [28] and international studies [7,29]. The mechanisms by which NPs may contribute to shorter hospital stays can be explained by their role of optimizing in-hospital care. Our findings indicate that NPs streamline care by ordering tests and procedures, managing medications, and performing daily patient assessments. This highlights the added value of employing NPs in busy surgical teams, where surgeons are primarily occupied in the operating room. In their absence, routine care may be delayed, affecting both length of stay and overall care quality [6].

The specific ways in which NPs enhance efficiency within inpatient teams are yet to be realized [30]. To our knowledge, no previous studies have reported on the specific interventions performed by NPs in surgical setting, except for a clinical report from the Division of Pediatric Neurosurgery. That report described NP activities, across all surgical phases, including discharge planning, coordinating of rehabilitation, and scheduling follow-up visits [31].

### Limitation

Several potential limitations should be acknowledged when interpreting the study findings. First, this is a single medical center study, and results should be further replicated. Second, we did not perform randomization. Third, we did not assess differences between the teams themselves and the quality of care they provided (e.g., the quality of care and the quality of explanations given by the entire team), which may also affect the results. Yet our comparative design was of two similar surgical units, one with and one without a NP. Fourth, some processes that may have affected the outcomes were outside the scope of our study. For example, changes in the types of procedures performed and the severity of patients’ conditions, between periods and between units, may play an important role but could not be accounted for. Due to organizational constraints, the data we were able to extract from the medical files had information only on the type of surgery that the patient underwent and other potential confounders, such as disease severity, were missing. Fifth, the use of self-reported measures may introduce bias, such as recall bias or social desirability. Although validated instruments were used, patients’ responses may have been influenced by subjective perceptions or a desire to provide favorable answers. Finally, differences in the type of care provided may be affected by differences in managerial or organizational attributes of each unit, which operates each unit under separate management. However, the units had similar surgical case mix (types of operations), shared institutional environment, and comparable patient characteristics (e.g., age, sex, and surgical indication), which supports the rationale for comparison. Future studies should include other patient characteristics that reflect the overall complexity of the patient’s condition. Nonetheless, this is the most comprehensive comparative study to date to be conducted on NP role implementation and outcomes, from which lessons on NP roles can be drawn.

## 5. Conclusions

NPs are authorized to perform a wide range of care interventions across the pre-operative, in-hospital, and post-discharge phases. This study adds to current knowledge on the types of care that are actually performed by a nurse specialist. Moreover, our study provides evidence that patients cared for by an NP have lower levels of in-hospital anxiety, indicating the added value of NPs in the provision of person-centered care, above and beyond aspects more commonly under scrutiny, such as healthcare system efficiency and effectiveness. Future studies should further corroborate these findings in controlled research designs. The findings underscore the relevance of NPs in surgical settings and their association with improved patient-centered outcomes. To strengthen their integration, healthcare systems should work toward defining NP roles more clearly, emphasizing their roles in preventing unwarranted effects such as in-hospital anxiety. Taken together, findings from this study showcase the value of introducing NPs roles into medical systems and their contribution to both patients and the healthcare system.

## Figures and Tables

**Table 1:** Baseline characteristics of patients in two units.

Variables	Total Sample (N = 315)	With NP (Unit A) (N = 156)	Without NP (Unit B) (N = 159)	*p*
Age Mean (SD)	49.52 (17.86)	45.6 (18.56)	54.15 (15.88)	<0.01
Sex				
Female	174 (54.5%)	94 (62.7%)	70 (46.4%)	<0.01
Male	137 (45.5%)	56 (37.3%)	81 (53.6%)	
Family status				
Single	59 (19.7%)	32 (21.2%)	27 (18.1%)	0.79
Married	209 (69.7%)	103 (68.2%)	106 (71.1%)	
Others	32 (10.7%)	16 (10.6%)	16 (10.7%)	
Ethnicity				
Jewish	98 (32.8%)	38 (25.3%)	60 (40.3%)	0.01
Muslim	89 (29.8%)	46 (30.7%)	43 (28.9%)	
Others	112 (37.5%)	66 (44%)	46 (30.9%)	
Religiosity				
Secular	134 (46.2%)	69 (47.9%)	65 (44.5%)	0.76
Traditional	97 (33.4%)	48 (33.3%)	49 (33.6%)	
Religious	59 (20.3%)	27 (18.8%)	32 (21.9%)	
Education				
Up to 12 years	212 (71.6%)	117 (79.1%)	95 (64.2%)	0.03
12 years or more	84 (28.4%)	31 (20.9%)	53 (35.9%)	
Type of Surgery				
Hernia Surgery	82 (30.4%)	31 (20.9%)	51 (41.8%)	<0.01
Bariatric Surgery	81 (30%)	69 (46.6%)	12 (9.8%)	
Cholecystectomy + appendectomy	107 (39.6%)	48 (32.4%)	59 (48.4%)	

NP = nurse practitioner; SD = standard deviation.

**Table 2:** Interventions performed by the NP for each patient.

Pre-Operative (N, %)	
Coordination of services	109 (34.6%)
Patient counseling	54 (17.1%)
Admission to the unit	6 (1.9%)
Preparations for operation	136 (43.2%)
Sum of pre-op activities (mean, SD)	2.14 (0.73)
**In-Hospital**	
Admission from the operating room	22 (7%)
Daily check-up	121 (38.4%)
Coordination of services	127 (40.3%)
Test orders	62 (19.7%)
Consultation orders	82 (26%)
Special procedures	27 (8.6%)
Medication management	139 (44.1%)
Sum of in-hospital activities (mean, SD)	3.86 (1.25)
**Post-Hospital**	
Discharge preparations	94 (29.8%)
Preparing the discharge letter	3 (1%)
Outreach (phone follow-up)	0
Coordination of post-discharge visit	1 (3%)
Sum of post-hospital activities (mean, SD)	1.03 (0.17)
**Total Sum of activities (mean, SD)**	6.51 (2.03)

**Table 3:** Outcome measures.

Symptoms (Mean, SD)	With NP (Unit A)	Without NP (Unit B)	*p*-Value
Pain			
Pain interference in hospital	3.61 (2.46)	4.15 (2.46)	0.06
Pain interference after discharge	0.89 (1.44)	1.21 (1.78)	0.02
Pain interference difference between in-hospital and discharge	−2.78 (2.97)	−2.56 (2.74)	0.57
Pain severity in hospital	4.53 (2.08)	4.60 (2)	0.76
Pain severity after discharge	1.13 (1.54)	1.39 (1.72)	0.24
Pain severity difference between in-hospital and discharge	−3.53 (2.55)	−2.99 (2.31)	0.1
Anxiety			
HADS in hospital	3.52 (4.37)	5.42 (5.06)	<0.001
HADS after discharge	1.81 (3.22)	2.26 (3.43)	0.32
HADS difference between in-hospital and discharge	−1.77 (6.05)	−3.04 (7.01)	0.06
In-hospital care			
LOS (days)	2.83 (1.24)	3.70 (3.85)	0.001

*p*-value derived from Fisher’s exact test. LOS = length of stay; SD = standard deviation; HADS = Hospital Anxiety and Depression Scale.

**Table 4:** Multivariable linear regression for the prediction of pain, anxiety, and length of stay.

	Post-Hospitalization Pain Interference		In-Hospital Anxiety		Length of Stay	
	β	*p*-value	β	*p*-value	β	*p*-value
Patients without an NP (unit B) (ref: patients with NP, unit A)	0.07	0.43	0.23	0.001	0.09	0.22
Age	0.16	0.05	0.11	0.1	0.12	0.05
Sex: Female (ref: Male)	0.06	0.49	0.15	0.03	−0.11	0.13
Ethnicity: Jews (ref: non-Jews)	−0.38	0.65	−0.04	0.52	0.08	0.25
Years of education: over 12 years (ref: less than 12 years)	−0.16	0.04	0.05	0.41	0.09	0.14
Surgery type (ref: GI)						
Bariatric	0.1	0.28	−0.08	0.29	0.01	0.83
Hernia	0.24	0.62	−0.20	0.01	−0.09	0.25

NP = nurse practitioner, GI = gastrointestinal: Cholecystectomy and appendectomy.

## Data Availability

The data that support the findings of this study are not publicly available due to ethical and privacy restrictions. Survey data were collected using Qualtrics, a secure web-based platform accessible only to the research team through individual login credentials. All participant identifiers were coded, and written informed consent forms are stored securely by the principal investigator.

## Public Involvement Statement

No public involvement in any aspect of this research.

## Guidelines and Standards Statement

This manuscript was drafted against the Strengthening the Reporting of Observational Studies in Epidemiology (STROBE) guidelines for observational research.
